# Breast carcinoma with choriocarcinomatous features: a case report and review of the literature

**DOI:** 10.1186/1477-7819-12-239

**Published:** 2014-07-30

**Authors:** Yanyun Zhu, Mei Liu, Jinyu Li, Fangfang Jing, Ruixia Linghu, Xiaoqin Guo, Shunchang Jiao, Junlan Yang

**Affiliations:** 1 Department of Medical Oncology, Chinese PLA General Hospital, 28 Fuxing Road, Haidian District, Beijing 100853, China; 2 Department of Pathology, Chinese PLA General Hospital, 28 Fuxing Road, Haidian District, Beijing 100853, China; 3 The First Out-patient Department, Bureau of Management and Social Security, Headquarters of the General Logistics Department of PLA, 22 Fuxing Road, Haidian District, Beijing 100842, China

**Keywords:** Breast cancer, Breast carcinoma with choriocarcinomatous features, Breast infiltrating ductal carcinoma, Chemotherapy, Human chorionic gonadotropin

## Abstract

**Background:**

Breast carcinoma with choriocarcinomatous features (BCCF) is a rare variant of breast cancer, characterized by high expression of human chorionic gonadotropin (HCG) in cancer cells such as multinucleated syncytiotrophoblast-like giant cells. The first case of BCCF was reported in 1981 by Saigo and Rosen. Only one case of BCCF was reported to show no component of breast ductal carcinoma, and only partially cancer cells, such as multinucleated syncytiotrophoblast-like giant cells, expressed HCG in all previous BCCF cases. Here, we report the first BCCF case without any component of breast ductal carcinoma in which HCG was found to express in all cancer cells.

**Case presentation:**

A 32-year-old female patient presented with a small lump in her left breast 3 years prior. The mass was clinically suspected to be breast infiltrating ductal carcinoma based on breast excisional biopsy and magnetic resonance imaging findings. Due to rupture and bleeding of the left kidney, the left kidney excisional biopsy was performed. After a retrospective analysis of the initial excised breast cancer and breast cancer metastatic to the kidney, the cancer cells were positive for HCG by immunohistochemistry, and multinucleated or mononucleated giant cells resembled syncytiotrophoblastic and cytotrophoblastic cells which could be seen in a background of poor differentiated breast carcinoma and extensive necrosis and hemorrhage in the lesion. Thus, a final diagnosis of BCCF and BCCF metastatic to the kidney was made. After combination of surgical resection (the affected left breast and left kidney wereremoved) and consecutive chemotherapy consisting of docetaxel, epirubicin, cisplatin, lobaplatin, and capecitabine, the patient achieved favorable therapeutic efficacy (the HCG level returned to normal values, the metastatic lesions in the lungs disappeared, and the survival was 37 months). Capecitabine was very efficient and highly recommended due to its superior efficacy in reducing the HCG level and eliminating the metastatic lesions in the lungs.

**Conclusions:**

This is the first report of a rare case of BCCF without any component of breast ductal carcinoma, featured by high expression of HCG in all cancer cells. Combination of surgery and chemotherapy (especially capecitabine) achieved a favorable therapeutic efficacy.

## Background

Breast carcinomas can produce various hormones normally not secreted by the breast, including human chorionic gonadotropin (HCG), human placental lactogen (HPL), adrenocorticotropic hormone (ACTH), and norepinephrine [1,2]. HCG has been detected in the serum in 12% to 33% of patients with breast carcinomas [3]. However, histologic examination of cancer did not reveal any evidence of choriocarcinomatous differentiation, nordid the clinical examination suggest any other source of HCG [2-4]. Breast carcinoma with choriocarcinomatous features (BCCF) is a rare variant of breast carcinoma and was first reported by Saigo and Rosen in 1981 [4]. BCCF is characterized by HCG-expressing highly atypical cancer cells morphologically similar to choriocarcinoma cells admixed with a malignant epithelial and/or mesenchymal component [5]. Most cases of BCCF have shown breast-infiltrating ductal carcinoma or ductal carcinoma in situ with choriocarcinomatous features [3,5]. Only one case of BCCF showed no component of breast ductal carcinoma [6]. In all previous BCCF cases, only some cancer cells, such as multinucleated syncytiotrophoblast-like giant cells, expressed HCG [3,5]. Here, we report a rare case of BCCF without any component of breast ductal carcinoma, featured by high expression of HCG in all cancer cells.

In this case report, the history, physical examination, laboratory findings, imaging studies, and pathological findings of BCCF in a 32-year-old woman are described and previous literatures about BCCF are reviewed.

## Case presentation

**Figure 1:**
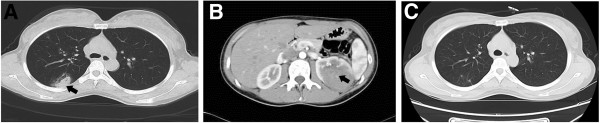
**CT (computed tomography) imaging of the lung and kidney of the patient.** Breast carcinoma with choriocarcinomatous features (BCCF) metastatic to the lung **(A)** and left kidney **(B)**. The metastatic lesions are indicated with black arrows. **(C)** The lesions in the lungs disappeared after a further 9 cycles of capecitabine.

**Figure 2:**
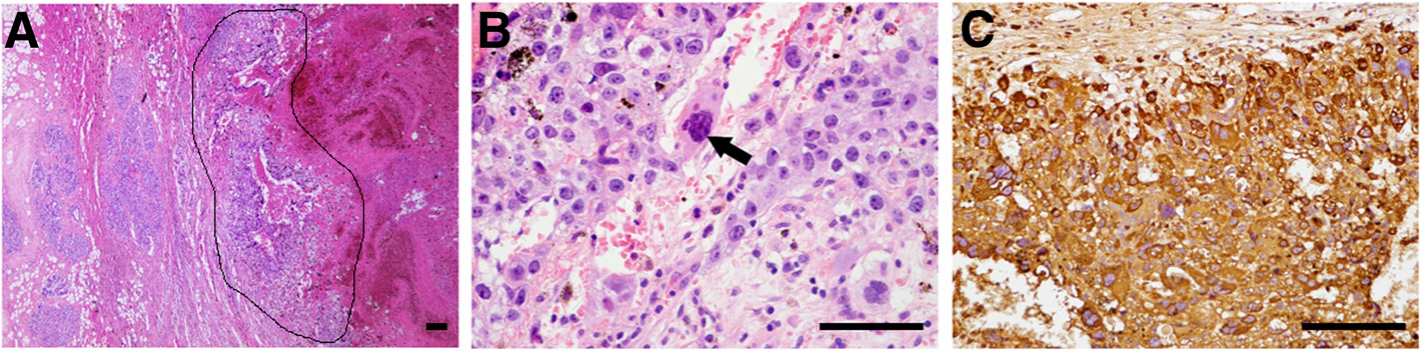
**Histology of breast carcinoma with choriocarcinomatous features (BCCF). (A)** Hematoxylin-eosin (HE) staining at a low magnification. The BCCF showed well-demarcated borders with extensive hemorrhage (the BCCF is indicated with black lines), and no infiltrating ductal carcinoma and ductal carcinoma in situ found. Error bars represent 100 μm. **(B)** Hematoxylin-eosin (HE) staining at a high magnification. A sheet-like arrangement of oval-shaped epithelial cells with prominent nucleoli was seen. Multinucleated giant cells (indicated by black arrows) with oval nuclei, prominent multiple nucleoli, and irregular chromatin clumping resembling syncytiotrophoblastic cells could be also be seen. Error bars represent 50 μm. **(C)** Immunohistochemistry (IHC) of human chorionic gonadotropin (HCG) demonstrates that all the cancer cells show strong HCG staining. Error bars represent 50 μm.

**Figure 3:**
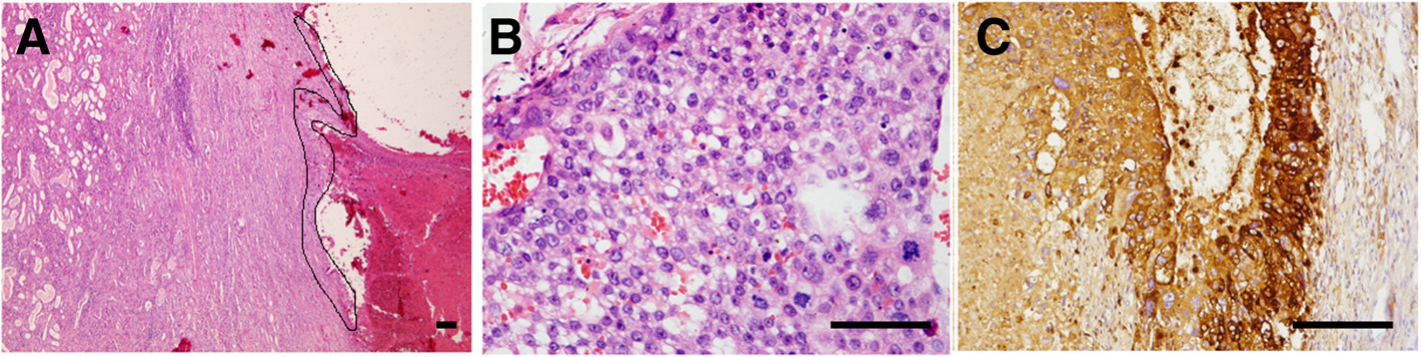
**Histology of breast carcinoma with choriocarcinomatous features (BCCF) metastatic to the kidney. (A)** Hematoxylin-eosin (HE) staining at a low magnification. The BCCF metastatic to the kidney (presented as a thin border of intact choriocarcinoma, indicated with black lines) is well-circumscribed and cystic, surrounded by normal renal tissue, with extensive hemorrhage around it. Error bars represent 100 μm. **(B)** HE staining at a high magnification. In the background of hemorrhage, giant cells with prominent pleomorphic nuclei, and abundant acidophilic and vacuolated cytoplasm resembling cytotrophoblastic cells could be seen. Error bars represent 50 μm. **(C)** Immunohistochemistry of human chorionic gonadotropin (HCG) demonstrates that all the cancer cells show strong HCG staining. Error bars represent 50 μm.

A 32-year-old woman, gravida 2 and para1, withregular menstruation, detected a small lump of 2 × 1.5 cm in her left breast upon self-examination in July 2010. The breast ultrasound confirmed the presence of a 1.8 × 1.2 cm lump of low echo-levels in August 2010. In January 2011, the position emission tomography-computer tomography (PET-CT) of the whole body revealed that there were metabolism-elevating occupying lesions in the left breast, left kidney, and two lungs. Computed tomography (CT) confirmed the occupying lesions in the lung and left kidney (Figure 1A,B). The core needle biopsy in the left breast was performed, but no cancer cells were detected. The levels of human chorionic gonadotropin (HCG) was 22,931 U/L (normal values: 0–5 U/L). In February 2011, the breast excisional biopsy was performed and a diagnosis of breast infiltrating ductal carcinoma was made. In the chemotherapy regimen, one cycle was 21 days. Two cycles of docetaxel (75 mg/m^2^, once per cycle) combined with epirubicin (75 mg/m^2^, once per cycle) reduced the lesions in the lungs and kidney and the HCG level (5,773 U/L at the end of the first cycle but 9,026 U/L at the end of the second cycle), while new lesions appeared in the lungs. The chemotherapy regimen was then changed to two cycles of docetaxel (75 mg/m^2^, once per cycle) and cisplatin (75 mg/m^2^, once per cycle), and the lesions in the lungs remained stable; the HCG level continued to decrease to 1,490 U/L at the end of the regimen. During this regimen, resection of the left kidney was performed due to rupture and bleeding. The left kidney excisional biopsy was also performed and a diagnosis of high-level infiltrating renal carcinoma (breast cancer metastatic to the kidney) was made. The chemotherapy regimen was changed to one cycle of docetaxel (75 mg/m^2^, once per cycle) and lobaplatin (35 mg/m^2^, once per cycle), and the HCG level continued to decrease to 57.86 U/L at the end of the regimen. Due to the severe marrow depression of docetaxel and lobaplatin, the chemotherapy regimen was changed to three cycles of docetaxel (75 mg/m^2^, once per cycle) and capecitabine (2 g/m^2^) (one cycle means once-daily administration for 2 weeks followed by 1 week of rest). At the end of the regimen, the HCG level continued to decrease to 17.64 U/L and the lesions in the lungs remained stable. A single capecitabine was then used instead of docetaxel and capecitabine. After two cycles of capecitabine (2 g/m^2^, one cycle means once-daily administration for 2 weeks followed by 1 week of rest), the HCG level continued to decrease to 0.12 U/L as didthe lesions in the lungs. The excised breast cancer and breast cancer metastatic to the kidney were retrospectively analyzed using HCG immunohistochemistry (IHC) staining. The IHC results demonstrated that all the cancer cells strongly expressed HCG, and the final pathological diagnosis was corrected to BCCF and BCCF metastatic to the kidney (Figures 2 and 3). After a further 9 cycles of capecitabine (2 g/m^2^, one cycle means once-daily administration for 2 weeks followed by 1 week of rest), the HCG level returned to normal values and the lesions in the lungs disappeared (Figure 1C). At this time, the patient was still alive (the survival was 37 months), and was undergoing her 26^th^ cycle of capecitabine (2 g/m^2^, one cycle means once-daily administration for 2 weeks followed by 1 week of rest) with no additional treatment.

### Pathologic findings

Grossly, the left breast of the patient was normal and BCCF did not protrude through the skin surface. The BCCF was 3.2 × 3.2 × 1.8 cm in size, and was a solid, well-circumscribed, and dark red mass, with extensive necrosis and hemorrhage in the lesion. The BCCF metastatic to the kidney was 5 × 3.5 × 2 cm in size, and was a solid, well-circumscribed, and chromatic mass, with extensive necrosis and hemorrhage in the lesion.Histologically, the BCCF showed well-demarcated borders with extensive hemorrhage, and no infiltrating ductal carcinoma and ductal carcinoma in situ could be found (Figure 2A). At high magnification, a sheet-like arrangement of oval-shaped epithelial cells with prominent nucleoli was seen. Multinucleated giant cells with oval nuclei, prominent multiple nucleoli, and irregular chromatin clumping resembling syncytiotrophoblastic cells could also be seen (Figure 2B). The BCCF metastatic to the kidney showed a very similar pattern in histology (Figure 3A,B). The cancer (presented as a thin border of intact choriocarcinoma) was well-circumscribed and cystic, surrounded by normal renal tissue, with extensive hemorrhage around it (Figure 3A). In the background of hemorrhage, giant cells with prominent pleomorphic nuclei, and abundant acidophilic and vacuolated cytoplasm resembling cytotrophoblastic cells could be seen (Figure 3B). In all, the BCCF and BCCF metastatic to the kidney were made up of proliferation of large sized cells with high nucleus/cytoplasm ratio and increased nuclear chromatin. Multinucleated or mononucleated giant cells resembling syncytiotrophoblastic and cytotrophoblastic cells could be seen. This finding was similar to choriocarcinoma originating from genital tract. No subtypes of breast infiltrating ductal carcinoma or ductal carcinoma were identified in the cancer. No lymphovascular invasion was identified.

IHC staining was performed on paraffin-embedded tissue sections, using a standard avidin-biotin-peroxidase complex method. The IHC staining demonstrated that all the cancer cells strongly expressed HCG in the BCCF and BCCF metastatic to the kidney (Figures 2C and 3C). In BCCF, other IHC markers were described as follows: HER-1 (++); HER-2 (-); p53(-); Cyclin D_1_ (-); ER (-); Ki-67 (+ >75%); PR (-); Top-II_α_ (+50% to 75%); p120 (++); CK5 (-); CK7 (+); CK20 (-); CK (+); EMA (+); and GCDFP-15 (+). In BCCF metastatic to the kidney, other IHC markers were described as follows: HER-2 (-); ER (-); Ki-67 (+ >75%); PR (-); CK (+); CK7 (+); CK20 (-); EMA (+); p63 (-); Vimentin (-), and PLAP (-).

According to the IHC study findings, the diagnosis of BCCF and BCCF metastatic to the kidney was confirmed.

## Discussion

BCCF is a rare variant of breast cancer, characterized by high expression of HCG in cancer cells such as multinucleated syncytiotrophoblast-like giant cells [5,7]. The origin of BCCF is not clear. Some researchers suggested that osseous and/or sarcomatoid metaplasia usually occurs in the development of breast infiltrating ductal carcinoma [3]. If metaplasia occurs in the breast infiltrating ductal carcinoma expressing HCG, the breast infiltrating ductal carcinoma cells will show choriocarcinomatous features [3]. Since the first BCCF was reported in 1981 by Saigo and Rosen [4], there have been 18 cases of BCCF reported (including thecase in our current study). Only one case of BCCF was reported to have no component of breast ductal carcinoma [6]. Furthermore, only partially cancerous cells, such as syncytiotrophoblastic cells or cytotrophoblastic cells-like giant cells, expressed HCG in all previous BCCF cases [3,5]. In our case of BCCF, no component of breast ductal carcinoma could be found, and HCG was found to be expressed in all cancer cells.

We summarized the characteristics of all the 18 cases in Table 1 and Table 2. In the 18 cases, the age of all patients ranged from 22 to 71 years (the average age is 46.5 years) and most tumors were located in the right breast (13/18), although several tumors were also located in the left breast (5/18); our patient presented with left-breast tumors only. The tumors ranged from 1 to 10 cm, and the metastatic tumors had the same histology as the primary tumors. Most cases (16/18) of BCCF presented with breast ductal carcinoma, whereas only two cases presented with no breast ductal carcinoma, including one case reported by Hematiet al. [6] and the case in our study. The pathological feature of BCCF is similar to that of choriocarcinoma in the female genital tract [5,7]. The cytopathologic characteristics of BCCF were hemorrhagic necrosis in the background and multinucleated bizarre giant cells resembling syncytiotrophoblasts in the chorion. Another characteristic is a sheet-like arrangement of oval-shaped epithelial cells with prominent nucleoli considered to be intermediate trophoblasts. The positive HCG staining contributes to the diagnosis of BCCF. The pathological feature of BCCF in our case is similar to that reported by Hematiet al. [6]; both cases presented with poor differentiated carcinoma, with no component of breast ductal carcinoma or other types of breast cancer, extensive necrosis and hemorrhage in the lesion, and multinucleated or mononucleated giant cells resembling cytotrophoblastic cells or syncytiotrophoblastic cells could be seen. The percentage of HCG-positive cancer cells varied in all the 18 cases. Murata et al. reported that the percentage of HCG-positive cancer in all cancer cells was estimated to be 2% to 3% [7], whereas a percentage of ~30% was also reported by Resetkova et al. [8]. In Hematiet al.’s case, only somecancer cells were found to express HCG [6]. It is noteworthy that, in our case, approximately 100% of the cancer cells expressed HCG. However, the mechanism underlying the high expression of HCG in our case remains to be elucidated.

**Table 1:** Summary of reports in literature

**Patient**	**Age (years)**	**Localization**	**Size (cm)**	**LN**	**IDC-DCIS**	**HCG**	**HPL**	**CK**	**HER-2**	**Reference**
1	32	L	3.2	–	–	+	/	+	–	Present study
2	38	R	5	44/44	+	+	/	+	/	[7]
3	50	R	7	0/20	+	+	/	+	/	[9]
4	32	R	/	/	/	+	/	/	/	[10]
5	56	R	3.5	–	+	+	/	+	/	[11]
6	38	R	1.0	/	–	+	/	+	–	[8]
7	54	R	10	–	+	+	/	+	–	[8]
8	59	R	2.5	4/19	–	+	+	+	+	[3]
9	48	R	2.5	0/16	+	+	+	+	+	[3]
10	58	R	4	12/19	+	+	+	+	–	[3]
11	49	R	1.6	–	+	+	+	+	–	[3]
12	55	L	2.5	–	+	+	/	/	/	[4]
13	71	R	2.5	20/21	Mucoid	+	/	/	/	[12]
14	22	L	4	/	+	+	/	/	/	[13]
15	53	L	3.5	0/10	+	+	/	/	+	[14]
16	50	R	4	0/19	+	+	/	/	–	[14]
17	31	L	/	/	+	+	/	+	–	[15]
18	41	R	3	3	–	+	/	/	/	[5]

**Table 2:** Summary of reports in literature (continued)

**Patients**	**ER**	**PR**	**Ki-67**	**EMA**	**PLAP**	**P53**	**Follow-up**	**Metastasis**	**Treatment**	**References**
1	–	–	+	+	–	–	DFS	Lung, kidney	S + C	Present study
2	–	–	/	+	+	/	Died	Lung, chest wall, liver	S + C + R + E	[7]
3	+	–	/	/	/	/	/	No	S	[9]
4	/	/	/	/	/	/	Died	Left parietal lobe	C + R	[10]
5	–	+	+	/	–	/	DFS	No	S	[11]
6	–	–	/	100	/	/	DFS	No	S + C	[8]
7	–	–	/	/	/	/	Died	Back neck, Pelvis, lungs	S + C	[8]
8	–	–	+	17	/	–	Lost	/	S + C + R	[3]
9	–	–	+	40	/	+	DFS	No	S + C + R	[3]
10	+	–	–	10	/	+	DFS	No	S + C + R	[3]
11	–	–	–	2	/	–	Lost	/	S	[3]
12	/	/	/	/	/	/	Died	Lung, Lymph nodes	S	[4]
13	/	/	/	/	/	/	DFS	Lymph nodes	S + E	[12]
14	/	/	/	/	/	/	/	Lungs, skin	C	[13]
15	–	–	+80%	/	/	–	DFS	No	S	[14]
16	–	–	+80%	/	/	+	DFS	No	S	[14]
17	–	–	/	/	/	/	/	/	S + C	[15]
18	/	/	/	/	/	/	Died	Lung, Liver, Kidneys	C	[5]

“+”, Positive; “–”, Negative; “/”, Unknown. “Lost”, Lost to follow-up.

L, Left breast; R, Right breast; LN, Lymph node status; IDC-DCIS, Infiltrating ductal carcinoma-ductal carcinoma in situ; HPL, Human placental lactogen; HCG, Human chorionic gonadotropin; CK, Cytokeratin; ER, Estrogen receptor; PR, Progesterone receptor; EMA, Epithelial membrane antigen; PALP, Placental alkaline phosphatase; DFS, Disease free survival; S, Surgery; C, Chemotherapy; R, Radiotherapy; E, Endocrine therapy.

The diagnosis of BCCF is difficult, and should be discriminated from many other diseases. Initially, the first differential diagnoses were metastatic choriocarcinoma to the breast and poor differentiated anaplastic breast-infiltrating ductal carcinoma. Most patients with metastatic choriocarcinoma to the breast are pregnant and have a definite history of reproductive system tumors, and primary breast cancer could not be present in the breast cancer. However, acomponent of primary breast cancer, such as breast-infiltrating ductal carcinoma and/or ductal carcinoma in situ, could be commonly found in BCCF. Further, a positive staining of GCDFP-15 in BCCF also supports that BCCF is a primary breast cancer [10,13]. Although our case presented with a component of poor differentiated carcinoma without any component of breast ductal carcinoma, other evidence supports the diagnosis of BCCF: an immunohistochemical profile of GCDFP-15 (+), CK7 (+), CK20 (-), CK (+), CK5(-), EMA (+), 32-year-old female with regular menstruation and no history of reproductive system tumors,and no pregnancy during disease occurrence. Thus, our case should be diagnosis as BCCF. Furthermore, the differential diagnosis with poor differentiated anaplastic infiltrating ductal carcinomas mainly depends on the positive staining of HCG [5]. Another differential diagnosis is the primary renal tumor expressing HCG (usually high stage renal transitional-cell carcinoma), a very rare form of renal tumor [16]. However, in our case, no component of transitional-cell carcinoma was discovered, HCG was found to be expressed in both breast and kidney cancer, both of which showed a very similar pattern in histology. Further, animmunohistochemical profile of CK7 (+), CK20 (-), CK (+), and PLAP (-) in kidney cancer was found, supporting a diagnosis of the BCCF metastatic to the kidney.

BCCF is a highly malignant breast cancer with a poor prognosis. Most patients die from multiple metastases within a few months [7,8]. However, two of four patients reported by Erhan et al. were disease-free 2 and 4 years after diagnosis, respectively [3], and BCCF patients who were disease-free 1 year after surgery have also been reported [6,10]. The etiology behind the poor prognosis of BCCF still remains to be elucidated, although a possible mechanism is described as follows: pregnancy-associated proteins such as HCG act as immunosuppressive reagents, and make cancer cells in BCCF invade the immune defense of the host immune system, causing BCCF to become a highly invasive cancer [17-19]. The patient in our case underwent resection of both BCCF and BCCF metastatic to the kidney, and the serum level of HCG is normal. Thus, HCG might not play an immunosuppressing role in the presentcase.

Current therapeutic strategies for BCCF mainly consist of endocrine therapy, surgery, and chemotherapy. So far, most cases of BCCF presented with double negative staining of estrogen and progesterone (our case also presented with double negative staining of estrogen and progesterone), whereas only one case was estrogen-positive and one case was progesterone-positive [3,11]. Tamoxifen-based endocrine therapy was ineffective in treating BCCF patients, whereas other endocrine therapies, such as gonadotropin-releasing hormone analogues, are also ineffective [5,7,12]. In all, there has not been effective endocrine therapy towards BCCF so far. Surgery is generally considered to be effective in treating BCCF. Although there are BCCF patients who presented with multiple metastases and a short survival period after surgery [7,10], the BCCF patients who had a disease-free survival period of more than 1 year all underwent surgical resection [3,8,9,14]. The two patients reported by Erhan et al. who were disease-free 2 and 4 years after diagnosis also underwent surgical resection [3]. The chemotherapy in BCCF regimen still remains unclear [5,8,15]. Fluorouracil or etoposide, methotrexate, vincristine, and doxorubicin are reported to be effective in treating BCCF [7,10], whereas methotrexate, actinomycin D, and cyclophosphamide are reported to be ineffective [13]. Several studies suggested that BCCF patients received postoperative chemotherapy but their efficacy was not reported [3,8]. For the BCCF patients who could not receive surgery, chemotherapy has proved both effective [10] and ineffective [13]. In our case, the combination of surgery and chemotherapy was used in treating BCCF, achieving a favorable therapeutic efficacy (the HCG level returned to normal values, the metastatic lesions in the lungs disappeared, and the survival was 37 months). In our chemotherapy regimen, capecitabine may play an important role in achieving favorable therapeutic efficacy. The reason why capecitabine exerts superior therapeutic efficacy towards BCCF is described as follows. In common chemotherapy regimens for the treatment of choriocarcinoma, 5-fluorouracil is recognized as an effective drug, and capecitabine is an oral prodrug of 5-fluorouracil [20,21]. In the tumor, capecitabine is selectively activated and exertsa therapeutic effect towards tumors [21]. Thus, capecitabine may also exert a superior therapeutic efficacy towards BCCF, which is breast carcinoma with choriocarcinomatous features.

Thus, combination of surgery and chemotherapy (especially capecitabine) was recommended in treating BCCF.

## Conclusions

This is the first report of a rare case of BCCF without any component of breast ductal carcinoma, featured by a high expression of HCG in all cancer cells. A combination of surgery and chemotherapy (especially capecitabine) achieved favorable therapeutic efficacy (the HCG level returned to normal values, the metastatic lesions in the lungs disappeared, and the survival was 37 months).

## Consent

Written informed consent was obtained from the patient for publication of this case report and any accompanying images. A copy of the written consent is available for review by the Editor-in-Chief of this journal.

## Abbreviations

ACTH: Adrenocorticotropic hormone; BCCF: Breast carcinoma with choriocarcinomatous features; CK: Cytokeratin; CT: Computed tomography; EMA: Epithelial membrane antigen; ER: Estrogen receptor; GCDFP-15: Gross cystic disease fluid protein 15; HCG: Human chorionic gonadotropin; HE: Hematoxylin-eosin; HPL: Human placental lactogen; IDC-DCIS: Infiltrating ductal carcinoma-ductal carcinoma in situ; IHC: Immunohistochemistry; LN: Lymph node status; PALP: Placental alkaline phosphatase; PET-CT: Position emission tomography-computer tomography; PR: Progesterone receptor.

## Competing interests

The authors declare that they have no competing interests.

## Authors’ contributions

All authors have contributed substantially to the study. YZ, ML, JL, FJ, RL, and XG contributed to the design of the study, analysis of data, and writing of manuscript. JY and SJ contributed to the conception and design of the study. All authors read and approved the final manuscript.
